# HIV-Infected Adolescent, Young Adult and Pregnant Smokers: Important Targets for Effective Tobacco Control Programs

**DOI:** 10.3390/ijerph10062471

**Published:** 2013-06-18

**Authors:** Gerome Escota, Nur Önen

**Affiliations:** Division of Infectious Diseases, School of Medicine, Washington University, 660 South Euclid Avenue, Saint Louis, MO 63110, USA; E-Mail: nonen@dom.wustl.edu

**Keywords:** tobacco, smoking, HIV, AIDS, adolescent, young adult, pregnancy

## Abstract

Tobacco use is inextricably linked to a number of health risks both in the general and HIV-infected populations. There is, however, a dearth of research on effective tobacco control programs among people living with HIV, and especially among adolescents, young adults and pregnant women, groups with heightened or increased vulnerability secondary to tobacco use. Adolescents and young adults constitute a growing population of persons living with HIV infection. Early and continued tobacco use in this population living with a disease characterized by premature onset multimorbidity and chronic inflammation is of concern. Additionally, there is an increased acuity for tobacco control among HIV-infected pregnant women to reduce pregnancy morbidity and improve fetal outcome. This review will provide an important summary of current knowledge of tobacco use among HIV-infected adolescents, young adults and pregnant women. The effects of tobacco use in these specific populations will be presented and the current state of tobacco control within these populations, assessed.

## 1. Introduction

It is estimated that over 1.1 million people are living with HIV/AIDS in the United States [1] of whom 25% are women and 6% adolescents and young adults aged 13–24 years [2]. In 2009, 20% of newly diagnosed HIV-infected persons were aged 13–24 years [3]. Among women infected with HIV, approximately 75% are estimated to be in the reproductive age group (13–44 years) [4]. The mortality rate associated with HIV infection has significantly declined since the introduction of highly active antiretroviral therapy (HAART) [5,6], although still remains higher than that of the general population and life expectancy slightly lower [7,8,9]. HIV-infected persons in contemporary outpatient care are more likely to die from non-AIDS-defining illnesses (NADI) such as cardiovascular and cancer-related deaths, than AIDS-defining illnesses [10,11]. The occurrence of NADIs across all CD4 cell counts and level of viral suppression [12] suggests that other mechanisms apart from immunosuppression contribute to this excess mortality. The chronic inflammation associated with HIV infection has been thought to significantly contribute to the development of NADIs [13,14,15]. Co-infection with hepatitis C, viral latency, and microbial gut translocation are some of the conditions that contribute to HIV-associated persistent inflammation [16]. Regardless, behavioral factors play a fundamental role, and of those, tobacco use may be among the most important. 

At present, tobacco use is the leading cause of preventable mortality and morbidity and premature death in the United States [17,18]. It is also a major cause of preventable infant mortality and morbidity in the US and other industrialized countries [19]. Among HIV-uninfected adults, the prevalence of tobacco use is 19% (Table 1) and more common among those aged 18–44 years, without a Bachelor’s degree, who are uninsured, and who belong to poorer families [18,20]. The prevalence of tobacco use is 50–70% in the HIV-infected population, 2–3 times higher than the general population [21,22] (Table 1), and is also more common among younger persons (median age 35 years) [23] with lower socio-economic status [24].

**Table 1:** A comparison of the prevalence of tobacco use between HIV-infected and general population.

Groups	General population	HIV-infected
Adults	19% [ 20]	50–70% [ 21,22]
Adolescents/young adults	23% [ 18]	4% (HIV acquired vertically) [ 25] 39% (HIV acquired behaviorally) [26] 21% (over-all estimate) [27]
Pregnant women	5–36% (over-all 14%) [ 28]	40–54% (before 1998) [29,30] 14% (after 1997) [31]

There is now evidence to suggest that the use of tobacco among adolescents and young adults is associated with development of respiratory and cardiovascular problems that serve as precursors to more long term and chronic diseases in late adulthood [18]. Thus, the higher prevalence of tobacco use among HIV-infected adolescents and young adults is alarming as HIV infection, itself, has also been associated with early-onset multimorbidity [32]. Tobacco use during pregnancy is associated with significant maternal and fetal morbidity and mortality. Furthermore, tobacco use increases the risk of vertical transmission of HIV infection, independent of maternal disease status and use of other substances [30]. Therefore, there is a critical need to address tobacco use among these vulnerable HIV-infected groups and to ensure that effective tobacco control strategies are implemented.

At present, the combination of behavioral modification through counseling and pharmacotherapy is the mainstay of tobacco control programs in the general population [33]. There are a dearth of studies that support the effectiveness of these well-established strategies among persons living with HIV/AIDS. In fact, there are currently no studies among HIV-infected adolescents and pregnant women. The studies among HIV-infected adults that have been published so far have found that these conventional approaches to tobacco control are suboptimal in the HIV-infected population [34]. HIV-infected persons frequently have additional comorbidities that make tobacco cessation particularly challenging, including alcohol and illicit drug use and psychological stressors [35]. Failure to promote an effective tobacco control program in this high risk population is concerning as the mortality and morbidity benefits of tobacco cessation are undisputable [36]. Among persons living with HIV/AIDS, cessation of tobacco use was associated with a reduction in the adjusted incidence rate ratio for the development of cardiovascular disease from 2.32 to 1.49 after only three years of tobacco abstinence [37]. In the general population, tobacco cessation during pregnancy prevented 5–6% of perinatal deaths, 7–10% of preterm deliveries, and 17–6% of low-birthweight births [36].

In this review, we first examine the epidemiology of tobacco use among both HIV-infected and uninfected adolescents, young adults and pregnant women. We then review the impact of tobacco use among HIV-infected persons and discuss different tobacco control strategies that have been studied. Next we look at the current state of tobacco control among adolescents, young adults and pregnant women in the general population. Finally, we examine how tobacco control can be addressed among HIV-infected adolescents, young adults and pregnant women, applying lessons derived from studies in the HIV-uninfected population.

## 2. Epidemiology of Tobacco Use among Adolescents and Young Adults

### 2.1. General Population

Current estimates show that 88% of adult smokers in the US initiate tobacco use before 18 years [38] and 99% before 26 years [18]. Experimentation with tobacco peaks at age 11 to 13 years among adolescents and more than one third become regular habitual smokers thereafter [39]. In 2009, 23% of US high school students were current smokers [18] (Table 1). Among all age groups in the US, the highest prevalence of tobacco use is among young adults aged 18–25 years with a rate of 34% [18]. In general, these estimates among adolescents and young adults declined from the late 1990’s but the rate of decline considerably diminished in the last few years. Prevalence has remained highest among American Indians and Alaskan Natives, and among those with low socioeconomic status [18].

### 2.2. HIV-Infected Population

At present, there are only a few studies that address the prevalence of tobacco use among HIV-infected adolescents. The Reaching for Excellence in Adolescent Care and Health Project was a multicenter collaboration in the US that enrolled both HIV-infected and uninfected adolescents aged 12–18 years from 1996 to 2000 [26]. HIV infection among infected adolescents was acquired through high risk behavior (*i.e.*, sexual activity, intravenous drug use) and those with vertically-transmitted HIV infection were excluded. HIV-uninfected adolescents with similar high risk behaviors were likewise recruited for comparison. In this cohort of 325 HIV-infected adolescents (26% male, 73% black), the over-all prevalence of tobacco use was 39% (Table 1), of whom, 27% were daily and 11% were weekly smokers. Fifty eight percent of HIV-infected males used tobacco (39% daily and 19% weekly smokers) while only 34% of females smoked (25% daily and 9% weekly smokers). The over-all prevalence of tobacco use among HIV-uninfected counterparts was also high at 36%. The rate of tobacco use among males was lower (42%) but among females, the rate was comparable (34%) to the HIV-infected group. 

The prevalence of tobacco use among adolescents with vertically transmitted HIV infection is generally lower. In a cohort of 206 HIV-infected adolescents aged 9–16 years in New York, 4% used tobacco (Table 1) compared with 7% of HIV-uninfected adolescents (n = 134) matched by age and demographics (*p* = 0.97) [25]. In a recent study of 266 persons aged 2–25 years, with either vertically or horizontally acquired HIV infection, the over-all prevalence of tobacco use was 21% (68% male, 93% black) [27] (Table 1). Subjects who used tobacco in this study were all between 18–25 years.

Lastly, data demonstrate a median age of 16–18 years at the time of tobacco initiation in the HIV-infected population [40,41]. 

## 3. Epidemiology of Tobacco Use among Pregnant Women

### 3.1. General Population

The proportion of pregnant women in the general population who uses tobacco varies by state and ranges from 5% to 36% (over-all prevalence of 14%) [28] (Table 1). These estimates are plagued by underreporting and nondisclosure when self-report is compared to biochemical markers of tobacco use [42,43]. Hence, the true prevalence of tobacco use in the pregnant population is expected to be higher. Similar to adolescents and young adults, the prevalence is higher among younger women aged <25 years, American Indians and Alaskan Natives, and those with lower socioeconomic status. Likewise, this estimate has progressively declined in recent years [44].

### 3.2. HIV-Infected Population

In the US, prevalence estimates for tobacco use among HIV-infected pregnant women have varied considerably. Estimates from the pre- and early HAART era ranged between 31–54% (Table 1). A survey of all liveborn deliveries from the New York State Medicaid data from 1988 to 1990 identified a 40% prevalence of tobacco use among HIV-infected women (n = 768) during pregnancy [30]. Tobacco users were more likely to be white, over the age of 25 years, and multiparous. In a separate cohort, the Pediatric AIDS Clinical Trials Group Study 185, a multicenter study conducted from 1993–1997 to investigate the use zidovudine and anti-HIV immunoglobulin in preventing mother-to-child HIV transmission, a 31% prevalence of tobacco use was observed [45]. On the other hand, the Women and Infants Transmission Study which enrolled a cohort of 634 pregnant women from different centers in the US from 1990 to 1998, documented a 54% prevalence in their cohort [29]. 

More recent estimates match current prevalence rates in the general population. In Florida, an administrative data review of all HIV-infected women who delivered between 1998 and 2007 demonstrated a 14% prevalence of tobacco use during pregnancy [31] (Table 1). Univariate analysis showed that pregnant smokers were more likely to be black, less than 35 years old, unmarried, and have no high school degree. In the Pediatric HIV/AIDS Cohort Study, an 18% prevalence of tobacco use was observed among 480 HIV-infected pregnant women enrolled from different centers in the US [46]. This study also found that self-report of tobacco use had an 86% correlation with meconium cotinine levels.

It is not known why prevalence estimates of tobacco use are higher during the pre- and early-HAART era. It likely reflects the downward trend in the prevalence rate seen also in the general population which is mainly a result of more intensive campaigns against tobacco use during pregnancy in recent years.

## 4. Impact of Tobacco Use in the HIV-Infected Population

**Figure 1:**
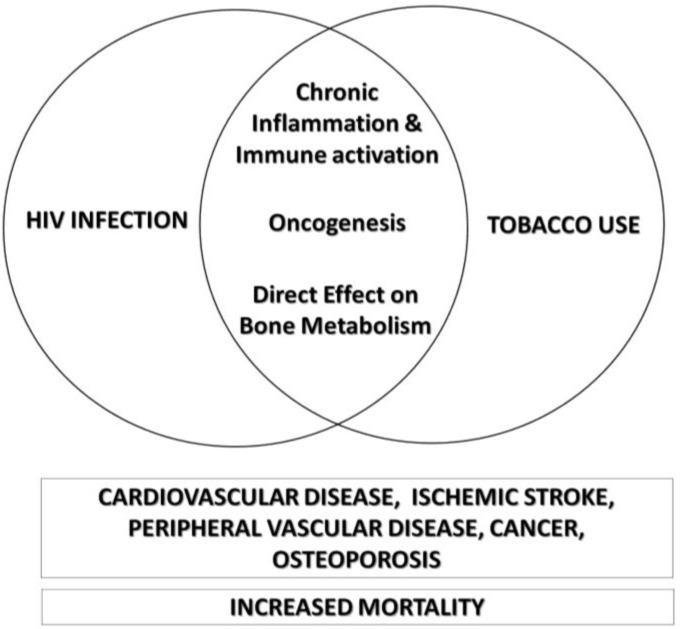
Through shared pathogenic mechanisms, tobacco use magnifies the independent association of HIV infection with cardiovascular disease, ischemic stroke, peripheral vascular disease, cancer, osteoporosis, and increased mortality.

Tobacco use and HIV infection both lead to increased mortality risk and predispose persons to developing cardiovascular disease [47,48], ischemic stroke [49,50], peripheral vascular disease [51], lung cancer [52], cervical cancer [53,54], and osteoporosis [55,56] independent of each other. Although definitely multifactorial, the pathogenesis of these complications reflects certain mechanisms shared by both HIV infection and tobacco use (Figure 1).

HIV infection is associated with persistent inflammation and immune activation that persist even after full virologic suppression and CD4 cell count recovery with HAART [57]. Elevated markers of inflammation and coagulation (*i.e.*, interleukin 6, C-reactive protein, soluble CD14, and D-dimer) are predictive of higher over-all mortality and cardiovascular events [58,59] and are also believed to contribute to the increased risk for atherosclerosis [60], osteoporosis [61], and cancer [62] among HIV-infected persons. Similarly, tobacco use also leads to chronic inflammation and platelet and leucocyte activation that directly facilitate atherogenesis [63], carcinogenesis [64], and development of osteoporosis. Furthermore, it has been shown that tobacco use heightens HIV-associated immune activation. Miguez-Burbano *et al.* demonstrated, on multivariate analysis, that HIV-infected smokers had demonstrably higher IL-6 levels than HIV-infected nonsmokers after adjusting for gender, use of HAART, stage of HIV disease and body mass index [65]. Our group also found significant association between elevated levels of T-cell activation (*i.e.*, CD8+ HLA-DR+ and CD4+ HLA-DR+ T lymphocytes) and current tobacco use among HIV-infected persons with fully suppressed HIV-RNA for at least 2 years [66]. 

Tobacco use is directly oncogenic and causes many known malignancies including lung, colorectal, head and neck, pancreatic, and uterine cancer [67]. Similarly, HIV infection has been demonstrated to also have some oncogenic potential. Witsuba *et al.* demonstrated that lung cancers from HIV-infected persons had significantly more frequent microsatellite alterations compared with sporadic lung cancers from HIV-uninfected persons [68]. Impaired immune surveillance secondary to HIV-associated immune dysregulation is also believed to contribute to this enhanced oncogenesis [69].

Tobacco use also poses additional health risks to HIV-infected persons (Table 2). These include an increased risk for infections such as oral candidiasis [70], genital warts [71], pulmonary tuberculosis [72] and bacterial pneumonia [70], plus human papilloma virus related-malignancies such as anal and cervical cancer [73]. Tobacco use has also been associated with decreased health-related quality of life. Among 585 HIV-infected persons, tobacco use, independent of CD4 count, AIDS diagnosis, educational level, injection drug use, gender and age, was associated with lower scores for general health perception, physical functioning, bodily pain, energy, role functioning, and cognitive functioning in the Medical Outcomes Survey Scale (MOS)-HIV questionnaire [74]. There are also data that suggest an association with tobacco use and clinical progression of HIV infection (*i.e.*, decline in CD4 count, virologic failure, occurrence of opportunistic infections), however, this finding is not duplicated by other studies [75]. 

**Table 2:** Other effects of tobacco use in the HIV-infected population.

1.	Increases risk for certain infections:
	Periodontal disease [76] Hairy cell leukoplakia [70,77] Oral candidiasis [70,78] Warts [79] Genital warts [71] Tuberculosis [72] Non-tuberculous mycobacteria [80]
2.	Increases risk for certain pulmonary diseases
	Bacterial pneumonia [70,78,81] Spontaneous pneumothorax [82,83]
3.	Increases risk for certain malignancies
	Cervical cancer [84] Anal cancer [73]
4.	Associated with increased risk of certain neuro-psychiatric symptoms
	Depression [85] AIDS Dementia Complex [78]
5.	Associated with over-all decreased quality of life [74,86]

### 4.1. Impact of Tobacco Use HIV-Infected Adolescents and Young Adults

In the general population, the disease risks associated with tobacco use among adolescents and young adults are similar to those in older persons. Current evidence suggests that the cardiovascular and respiratory risks associated with tobacco use are established early among adolescent smokers and serve as precursors to more chronic and serious problems later in adulthood [18]. Tobacco use among adolescents and young adults has been directly linked to subclinical atherosclerosis and increased carotid intimal thickness [87,88] as well as to early markers of vascular endothelial injury [89]. It has also been directly associated with decreased forced expiratory volume [90] and has been shown to be an independent predictor of asthma, respiratory symptoms such shortness of breath and wheezing, and decreased exercise tolerance among adolescent smokers [91]. Since these health risks accumulate over time, it is expected that tobacco use established at an early age is associated with more adverse outcomes. 

Tobacco use also serves as a harbinger for other high risk behaviors. For example, adolescents who use tobacco are eight and 22 times more likely to use alcohol and cocaine, respectively, and are more likely to engage in other risky behaviors such as unprotected sexual intercourse, and unhealthy dieting techniques (*i.e.*, ingesting dietary supplements, not eating for ≥24 h) [92,93]. Among adolescents with vertically-transmitted HIV infection, smokers were significantly more likely to engage in high risk sexual behavior including having multiple sexual partners and unprotected vaginal and anal intercourse compared to nonsmokers [25]. 

### 4.2. Impact of Tobacco Use among HIV-Infected Pregnant Women

Tobacco use during pregnancy is associated with preeclampsia, small for gestational age and low birth weight neonates, spontaneous pregnancy loss, stillbirth, preterm delivery, premature rupture of membranes, placenta previa, placental abruption, and congenital malformation [94]. In addition, tobacco use during and after pregnancy has been strongly associated with sudden infant death syndrome even after adjusting for multiple covariates including race, maternal age and parity [95]. At present, tobacco use during pregnancy is one of the leading causes of preventable infant mortality and morbidity in the US and other industrialized countries [19] and tobacco cessation during pregnancy is one of the strategies employed to curb infant mortality rates in these countries [96]. Maternal HIV infection has also been associated with adverse pregnancy outcomes including low birth weight, preterm birth, and intrauterine growth retardation independent of other behavioral risk factors [45]. 

With improved understanding of mother-to-child transmission of HIV infection, timely initiation of antiretroviral drugs, increased Caesarian delivery, and avoidance of breastfeeding when appropriate among infants exposed to mothers with HIV infection, vertical transmission rate has declined to only less than 2% [97] in industrialized countries, compared to around 25–30% without these interventions [98]. There are, however, studies that link tobacco use during pregnancy with increased mother-to-child transmission of HIV infection. Using vital statistics birth data from the New York State Medicaid HIV/AIDS Research Database, Turner *et al.* demonstrated among 768 HIV-infected pregnant women (42% white Hispanic, 41% black) a 45% increase in vertical transmission associated with the use of tobacco after adjustment for multiple risk factors that included illicit drug use, maternal clinical status, and delivery factors [30]. This risk may be attributed to higher incidence of premature rupture of membranes as well as to placental ischemia associated with tobacco use [99]. 

## 5. Tobacco Control in the HIV-Infected Population

The factors that negatively influence tobacco control in the general population include low socioeconomic status [100], existing psychiatric illness [101], and history of substance abuse [102]. Among people living with HIV/AIDS, these factors are more prevalent and often co-exist [24] and tobacco control has been a real challenge in this population. In a study of 184 HIV-infected tobacco users in San Francisco (82% male, 53% white), about two-thirds were unemployed and almost half had an annual income of below $10,000 [103]. In another study in the US, nearly half of 2,864 HIV-infected persons surveyed had at least one psychiatric disorder [104]. Among over 500 HIV-infected persons in our institution, approximately 25% had depressive or anxiety symptoms [105,106]. Furthermore, Cofrancesco *et al.* reported significantly higher rates of previous (86% *versus* 67%) and current (28% *versus* 16%) substance use among people living with HIV/AIDS compared with uninfected counterparts [107].

In a review of literature related to tobacco use and HIV/AIDS published from 1980 to 2008, Harris only found 6 studies on tobacco control interventions. The vast majority (88%) characterized the impact of tobacco use among people with HIV/AIDS [108]. This paper highlighted the exigency of conducting studies that aim to determine the best strategy to promote tobacco cessation in the HIV-infected population.

We independently reviewed PUBMED and found 12 studies, published since 2000, that evaluated different tobacco cessation strategies (Table 3). The majority of the studies were small and nonrandomized. All randomized controlled trials compared conventional tobacco cessation practices (*i.e.*, nicotine replacement therapy (NRT), provision of self-help materials or brief health care provider advice) with either cellular phone counseling (two studies), motivational intervention (three studies), or facilitated group treatment (one study). The abstinence rate was only significantly higher in the cellular phone counseling groups compared to control. There are only three studies, all small, nonrandomized and non-controlled, which have examined the effectiveness of newer pharmacotherapeutic agents, buproprion (one study) and varenicline (two studies), in the HIV-infected population. The results of these three studies were promising as the abstinence rate and medication adverse effect profile closely approximated those in the general population [109,110,111,112].

**Table 3:** Studies on tobacco cessation in the HIV-infected population.

Author	Year	Design	Intervention	Number of Subjects	Follow-up	Abstinence Rate	Measure of Abstinence
Wewers [113]	2000	Non-randomized	NRT, weekly in-person or telephone counseling, skills training *versus* self-help materials	Intervention: 8 Control: not stated	6 weeks and 8 months	Intervention: 62% at 6 weeks; 50% at 8 months Control: 0% 6 weeks; 0% at 8 months *P* value not provided	Biochemical
Lazev [114]	2003	Non-randomized	Cellular phone counseling	Intervention: 20 No control	2 weeks	75%	Self-report
Vidrine [40]	2006	Randomized	Cellular phone counseling plus usual care *versus* usual care (brief doctor advice, self-help materials, NRT)	Intervention: 48 Control: 47	3 months	Intervention: 37% Control: 10% *P* = 0.0059	Biochemical
Elzi [115]	2006	Non-randomized	Counseling, NRT *vs.* no intervention	Intervention: 34 Control: 383	12 months	Intervention: 38% ontrol: 7% Odds ratio: 6.2 (CI: 2.8–14.3)	Self-report
Pedro-Clotet [116]	2006	Non-randomized	Buproprion	Intervention: 21 No control	12 months	Intervention: 38%; no significant drug interactions	Self-report
Ingersoll [117]	2009	Randomized	Motivational intervention plus NRT *versus* NRT plus self-help	Intervention: 18 Control: 22	3 months	Intervention: 22%, no difference from the control; but reduced cigarette consumption by ½ per day and percent of smoking days by 41%	Biochemical
Lloyd-Richardson [118]	2009	Randomized	Motivational intervention plus NRT *vs.* NRT plus usual care (2 brief counseling sessions and self-help materials)	Intervention: 232 Control: 212	6 months	Intervention: 9% Control: 10% Not statistically significant	Biochemical
Tornero [119]	2009	Non-randomized	Varenicline	Intervention: 22 No control	6 months	Intervention: 24%; adverse events and abstinence rate comparable in the general population	Biochemical
Vidrine [41]	2012	Randomized	Cellular phone counseling plus usual care *vs.* usual care (NRT, self-help materials, brief advice from provider)	Intervention: 236 Control: 238	3 months	Intervention: 9% Control: 2% *P* = 0.005	Biochemical
Moadel [120]	2012	Randomized	Facilitated group treatment plus NRT *vs.* usual care (NRT and self-help materials)	Intervention: 73 Control: 72	3 months	Intervention: 19% Control: 10% *P* = 0.11	Biochemical
Cui [121]	2012	Non-randomized	Varenicline	Intervention: 36 No control	12 weeks	Intervention: 42%; adverse events and abstinence rate comparable in the general population	Biochemical
Manuel [122]	2012	Randomized	Motivational intervention plus NRT *versus* prescribed advise (self-help materials discussed with therapist) plus NRT	Intervention: 15 Control: 15	1 month	Intervention: 20% Control: 0% *P* = 0.067	Biochemical

NRT–nicotine replacement therapy; CI–confidence interval.

A few conjectures can be derived from Table 3. First, the combination of tobacco cessation counseling (in person or by telephone) and use of NRT is still effective among HIV-infected tobacco smokers [113,115]. Other proposed strategies (*i.e.*, motivational interview, facilitated group treatment) have not been found to add additional benefits compared to this usual approach. Second, the use of buproprion and varenicline is expected to yield similar abstinence rates with that in the general population. However, randomized controlled trials involving these newer pharmacotherapeutic agents are needed.

## 6. Tobacco Control among Adolescents in the General Population

Adolescent and adult smokers share some common barriers to tobacco cessation including nicotine dependence and depression. However, adolescent smokers also have some barriers to tobacco cessation that may be more influential to them than to adults. Parental smoking negatively influences smoking behavior among adolescents. A survey of 4,502 adolescents who lived in two-parent households showed that adolescents whose parents stopped smoking were one third less likely to be ever smokers than those whose parents were still smoking and that adolescent smokers were two times more likely to quit smoking if they had parents who quit [123]. Peer influence has also been demonstrated to greatly impact smoking behavior among adolescents. Transition to daily tobacco use by the 12th grade at school among 270 adolescents was associated with tobacco use among parents and close peers on multivariate analysis in one study [124]. Another study demonstrated that among adolescents who initiated smoking at an older age (13–14 years), tobacco use among peers was the major influence in contrast to those who initiated tobacco use at a much earlier age (11–12 years) where parental smoking was found to be more influential [125].

The strategies for tobacco control among adolescents and adults differ in some ways. Adolescent tobacco control programs rely heavily on addressing psychosocial factors that influence tobacco use among the youth. Thus, some programs are carried out with the participation of parents, schools and communities. Furthermore, adolescent tobacco control programs rely less on the use of pharmacotherapeutic agents like NRT [126]. 

The main strategy for tobacco control among adolescents has been counseling. Compared to usual care (*i.e.*, brief advice, self-help pamphlets) or no intervention, physician-delivered counseling is associated with a two-fold higher abstinence rate [126]. Despite this, it is estimated that only 42–55% of adolescents are counseled against tobacco use during clinic visits [127,128]. A meta-analysis found that five to eight counseling sessions were associated with significantly higher abstinence rates compared with control (5% increase). Nine or more counseling sessions did not increase this abstinence rate further [129].

Counseling facilitated in schools and other community settings by teachers, nurses, healthcare counselors and other volunteers, as exemplified by the Not-On-Tobacco (N-O-T) program sponsored by the American Lung Association [130], has been shown to be effective in promoting tobacco abstinence based on pooled data from four studies on N-O-T (odds ratio 1.77, 95% CI 1–3.11) [131]. A meta-analysis done by Sussman showed 4–5% higher abstinence rates associated with classroom and school-based counseling compared with controls [129].

Similar to the general adult population, a variety of theoretical models have been utilized in tobacco control counseling whether in the clinic, school, or community, including the transtheoretical model, motivational enhancement, cognitive behavioral therapy, and social-influence-oriented approach (Table 4). These models are frequently combined in tobacco control programs. A recent meta-analysis of 24 randomized controlled trials, comprising a total of 5,000 patients, showed superiority of the transtheoretical model (two studies analyzed) and motivational enhancement (11 studies analyzed) in achieving tobacco abstinence of at least six months (each with odds ratio of 1.7) [131]. Cognitive behavioral therapy (six studies analyzed), in this meta-analysis was not associated with a statistically significant result. This is in contrast with Sussman’s earlier meta-analysis [129] that showed cognitive behavioral therapy (17 studies analyzed) was associated with a statistically significant treatment effect of 3.9%. This study also showed that social-influence-oriented approach was also associated with a significantly higher abstinence rate compared with usual care.

**Table 4:** Theoretical models used in smoking cessation counseling.

Counseling models	Theory
Transtheoretical model of change [132,133]	Smokers are classified in different stages: precontemplation (not intending to quit), contemplation (intending to quit within the next 6 months), preparation (intending to quit within the next 30 days), and recent action (tobacco cessation). Tobacco cessation counseling is then tailored based on this. Providers help smokers advance to the next stage until they ultimately quit.
Motivational enhancement [126,134]	Advantages and disadvantages of tobacco use/cessation are determined together with smokers’ beliefs and values in order to identify any uncertainties that smokers may have. These uncertainties are then focused on and smokers are then guided in making a detailed quit plan.
Cognitive behavioral therapy [129]	Detailed instructions on cognitive-behavioral self-monitoring and coping skills regarding tobacco cessation are given (*i.e.*, reasons for tobacco use are determined and smokers are taught coping skills)
Social-influence-oriented approach [129]	Strategies aimed at resisting social influences promoting tobacco use are emphasized (*i.e.*, awareness of tobacco industry promotions and empowerment of teenagers to protest against them)

Among adolescents, signs and symptoms of nicotine dependence develop first and herald the onset of tobacco addiction [135]. Adolescents also have a higher risk of becoming nicotine dependent compared with adults [18]. NRT’s are safe and well-tolerated by adolescents however, a number of studies have not found its use to be beneficial in this population. Moolchan *et al.* [136], compared NRT plus behavioral therapy with behavioral therapy alone among 120 adolescent smokers and found no statistically significant difference in abstinence rate at three months after study completion in either arm. Similarly, 30-day tobacco abstinence rate was comparable among 100 adolescent smokers assigned randomly to receive either NRT plus cognitive behavioral therapy *versus* cognitive behavioral therapy alone [137]. A recent meta-analysis by Grimshaw *et al.* [131], found no pooled treatment effect associated with the use of NRT in adolescents. For this reason, the Agency for Healthcare Research and Quality (AHRQ) does not recommend the use of NRT to be part of routine tobacco control programs among adolescents [126]. 

Likewise, the use of buproprion, an atypical antidepressant and tobacco cessation aid, has not been validated among adolescents. In a study conducted in Austria, the duration of abstinence was statistically longer among 11 nicotine-dependent adolescents who received buproprion compared to 11 subjects who did not (78 *versus* 30 days) [138]. However, in a study conducted in the US among 211 adolescent smokers, abstinence rates at 10 and 26 weeks did not statistically differ between those who received both nicotine patch and buproprion and those who received nicotine patch alone [139]. Similarly, Muramoto *et al.* showed that buproprion was only associated with a significantly higher abstinence rate at six weeks but not at 26 weeks among 312 adolescent smokers [140]. Pooled data from a meta-analysis by Grimshaw *et al*, failed to demonstrate statistically significant treatment effect with the use of buproprion [131]. In Muramoto’s study, although generally safe, buproprion use was associated with more headache and cough *versus* placebo [140]. Two suicide attempts occurred in the buproprion group, one in a patient with depression and probable eating disorder. At present, the AHRQ does not endorse the use of buproprion for tobacco control among adolescents.

Other pharmacotherapeutic agents for tobacco cessation, such as varenicline, a nicotinic receptor partial agonist, have not been studied among adolescent smokers to date. A preliminary study on the safety of varenicline was conducted by Faessel *et al.* [141]. The majority of adverse events were similar to those found in adults; mild in intensity and included nausea, headache, and vomiting. None of the adverse events led to drug discontinuation or dose reduction. 

Tobacco control among adolescents is however, promising primarily because majority of adolescent smokers are willing to stop using tobacco. At least 77% of adolescent current smokers have made one or more serious tobacco cessation attempts [142]. Unfortunately, majority of these attempts are unplanned and unassisted [143] and hence, only 10% lead to sustained abstinence [144]. This rate of failed quit attempts, however, exceeds that in adults [143] and adolescent smokers enrolled in tobacco control programs are twice more likely to succeed than those who are not [129].

## 7. Tobacco Control among Pregnant Women in the General Population

Factors that are associated with continued tobacco use during pregnancy include lower educational attainment, long tobacco use history (*i.e.*, at least five years), having a partner who uses tobacco, poor coping skills, coexisting psychiatric problems, and exposure to secondhand smoke [94,145,146]. There are also barriers to tobacco cessation that may be unique to women in general that can certainly influence decision to quit; women in the luteal phase of menstruation tend to be more successful at tobacco cessation compared to women in the follicular phase [94]. 

The main strategy used to promote tobacco cessation among pregnant women is also counseling. In a meta-analysis of 72 randomized controlled trials [147], the estimated pooled risk reduction associated with any form of counseling during any stage of pregnancy, although low, was significant at 0.94 (95% CI 0.93–0.96). Counseling was also associated with reduced rates of low birthweight neonates (RR 0.83, 95% CI 0.73–0.95) and preterm births (RR 0.86, 95% CI 0.74–0.98) but not with neonatal intensive care unit admissions, very low birth weight neonates, stillbirths, perinatal or neonatal mortality.

Similar counseling models as discussed previously are also utilized among pregnant smokers with varied success rates. In the same meta-analysis cited previously [147], cognitive behavioral therapy was the most studied counseling model with 31 RCTs reviewed (RR 0.95, 95% CI 0.93–0.97), undoubtedly increasing the over-all risk reduction associated with counseling in general. Strategies that involved provision of financial incentives (e.g., vouchers amounting to at least $50 [148,149]) to participants who successfully stopped tobacco use produced the greatest risk reduction (RR 0.76, 95% CI 0.71–0.81). Although the use of financial motivation is promising, there are currently no studies on policy implications of undertaking such a strategy at the population level [147].

The use of NRT during pregnancy is controversial primarily due to teratogenicity concerns. In experimental studies, nicotine was capable of crossing the placenta as well as achieving concentrations in breast milk 2–3 times higher than in plasma [150]. In animal models, it has been associated with fetal central nervous system abnormalities, cardiac malformations, pulmonary hypoplasia, uteroplacental insufficiency, and sudden infant death syndrome [150,151]. In humans, it has been linked to negative birth outcomes as well. A Danish cohort study among 20,603 pregnant smokers was conducted to determine the effect of NRT during the first 12 weeks of pregnancy [152]. The prevalence of congenital malformation (especially musculoskeletal defects) was found to be statistically higher among pregnant women who used NRT compared to those who did not (relative prevalence ratio 1.61, CI 1.01–2.58). This result should, however, be interpreted with caution as the proportion of pregnant women who used NRT in this study was small (1.2%). Furthermore, the result may have been confounded by mismatched risk factors between groups [153]. Pollak *et al.* showed a probable link between nicotine exposure and negative birth outcomes when an interim analysis revealed a higher rate of negative birth outcomes among pregnant smokers who received NRT compared to those who did not [154]. Gaither *et al*, in another retrospective study, found that pregnant smokers who used NRT had twice the risk of having low birth weight babies (OR 1.95, 95% CI: 1.10–3.46) while those who did not use NRT had less than twice the risk (OR 1.31, 95% CI: 0.92–1.87) compared to nonsmokers [155]. The authors postulated that prescription of NRT was biased toward heavy pregnant smokers who may have also had difficulty with tobacco cessation. 

As a result of these studies, nicotine is currently classified as a category D drug (positive evidence of human fetal risk; maternal benefit may outweigh fetal risk in serious or life-threatening situations). Despite this, many groups, including the American College of Obstetricians and Gynecologists (ACOG) [156], recognize its utility especially among high risk pregnant women who fail to stop using tobacco after counseling. The ACOG states that NRT can be recommended to such women under close supervision and after comprehensive and informed discussion on the risk of tobacco use and NRT. This statement emphasizes the fact that the risk of continued tobacco use is evidently greater than the possible risk of nicotine to the fetus. On the same note, supporters of NRT use during pregnancy also argue that nicotine levels are similar or even lower in abstinent pregnant women using NRT compared with those who continue to smoke as nicotine metabolism is increased during pregnancy [151,157].

A pooled risk reduction of NRT for tobacco cessation did not reach statistical significance in a recent meta-analysis of six RCTs comprising 1,745 pregnant smokers (RR 1.33, 95% CI: 0.93–1.91) most likely due to low adherence rates to NRT (7.2 to 29%) [158]. Whether this low adherence is attributed to side effects or other factors was difficult to ascertain as those studies who reported side effects mentioned only 10–25% adverse events rates (e.g., headache, dizziness, fatigue, heartburn, nausea, vomiting). Nonetheless, the meta-analysis found no statistically significant difference in the rates of miscarriage, stillbirth, premature birth, or low birthweight deliveries between groups that used NRT and those that did not.

At present, there are no clinical trials on the use of buproprion or varenicline for tobacco cessation among pregnant women. Both are classified as category C drugs (animal studies show adverse fetal effects but no controlled human studies or no animal or human studies; weigh possible fetal risk *versus* maternal benefit). Prior studies on the safety of buproprion among depressed pregnant women found higher rates of spontaneous abortion among those who used buproprion compared to those who did not (15% *versus* 4%, *p* = 0.009) [159] while a later and larger study found no difference in the prevalence of congenital malformation between these two groups [160]. There are currently no human studies on varenicline use among pregnant women.

An important issue to address in all tobacco cessation programs undertaken among pregnant women is whether these interventions lead to a significant postpartum abstinence rate once the main motivation for tobacco cessation is past [161,162]. Relapse rates range from 67–93% within the first postpartum year [145]. In the recent meta-analysis by Lumley, the over-all benefit of intervention on abstinence rates was only significant during pregnancy and up to five months postpartum [147]. These results imply that women are cognizant of the harms of tobacco use during pregnancy but not of the ill effects of passive smoking to the baby [145]. Counseling of parents about tobacco cessation during pediatric visits has been shown to be effective. A study that involved 2,901 mothers of newborns showed that mothers who received counseling postpartum had higher quit rates and lower relapse rates compared to those who only received hospital tobacco cessation pamphlets [163]. The same promising results were seen in other studies [164,165]. Counseling parents about tobacco cessation is currently recommended by the American Medical Association as part of the routine pediatric visit [166].

Pregnancy offers a special opportunity for clinicians to frequently promote tobacco cessation as clinic attendances increase significantly during the antepartum and postpartum periods. Despite this, only half of obstetricians are estimated to address tobacco cessation during each visit and only 28% discuss actual tobacco cessation interventions [167]. In a meta-analysis by Lumley *et al.*, low intensity intervention consisting of physician-delivered verbal counseling alone was associated with a statistically significant risk reduction in continued tobacco use (RR 0.95, 95% CI: 0.93–0.96) [147].

## 8. Smoking among HIV-Infected Adolescents, Young Adults and Pregnant Women

### 8.1. Lessons Learned from the General Population

The average number of attempts at tobacco cessation increases with age and the majority of smokers stop using tobacco after several failed attempts [168]. Furthermore, it is estimated that mortality rates with tobacco cessation before the age of 35 years are similar to that of non-smokers [169]. Hence, it is crucial that tobacco cessation interventions are initiated as early as possible among adolescents, young adults and pregnant women.

Tobacco cessation interventions conducted among adolescents and pregnant women have generally been successful (Table 5). They mirror strategies in the adult population with the exception of pharmacotherapy. An appreciable proportion of both adolescents [142] and pregnant women [38] also desire to quit. Physician-delivered counseling during clinic visits has also been generally successful in these groups [126,147]. This is however an underutilized strategy as only at least half of obstetricians and pediatricians address tobacco cessation during office visits [127,128,168]. 

**Table 5:** Tobacco cessation strategies used in the general population.

Group	Main strategy	Treatment effect
Adults	Counseling plus pharmacotherapy (NRT/buproprion/varenicline)	OR 1.82 (95% CI: 1.66–2.00) *vs.* usual care [33] OR 1.4 (95% CI: 1.20–1.60) *vs.* pharmacotherapy alone [126] OR 1.7 (95% CI: 1.30–2.10) *vs.* counseling alone [126]
Adolescents	Counseling	OR 2.9 (95% CI: 1.47–4.35) *vs.* usual care [129]
Pregnant women	Counseling alone or counseling plus NRT	Counseling alone: RR 0.95 (0.93–0.97) *vs.* usual care [158] NRT + Counseling: RR 1.33 (95% CI: 0.93–1.91) *vs.* usual care [158]

NRT—nicotine replacement therapy; OR—odds ratio; RR—risk ratio; CI—confidence interval.

### 8.2. Addressing the Problem

A major obstacle in addressing this issue is the lack of studies on tobacco control among these vulnerable groups. Nevertheless, studies conducted in the general population can be extrapolated. The prevalence rates of tobacco use among HIV-infected adolescents, young adults and pregnant women are also considerably lower than that of older HIV-infected persons and approach that of the general HIV-uninfected adult population (Table 1). Therefore, addressing the problem of tobacco use among HIV-infected adolescents, young adults and pregnant women provides an early opportunity to stop the trajectory of tobacco use at its nascency in the HIV-infected population. HIV infection also enables clinicians to address tobacco use more frequently among infected adolescents, young adults and pregnant women as these groups are usually engaged in care and are seen more frequently in clinic compared to uninfected counterparts. Thus clinicians, including infectious disease providers, need to actively assess tobacco use among their patients, be knowledgeable about tobacco cessation interventions, and be able to explain to patients the added risk that tobacco use poses to them above that of non-HIV-infected smokers. Modern technology (*i.e.*, tablet computers, iPad, mobile devices) can help in assessing tobacco use. For instance, in our HIV clinics, we conduct a twice yearly risk and needs assessment using iPads. The assessment includes questions on tobacco use and the patient answers the questions while waiting for their clinician in the examination room. Once the assessment is complete, a real-time print-out is then provided to the clinician who will assess the patient. This practice provides useful information regarding tobacco use, among others, and ensures that risk behaviors are identified and addressed in a busy clinic setting. 

**Figure 2:**
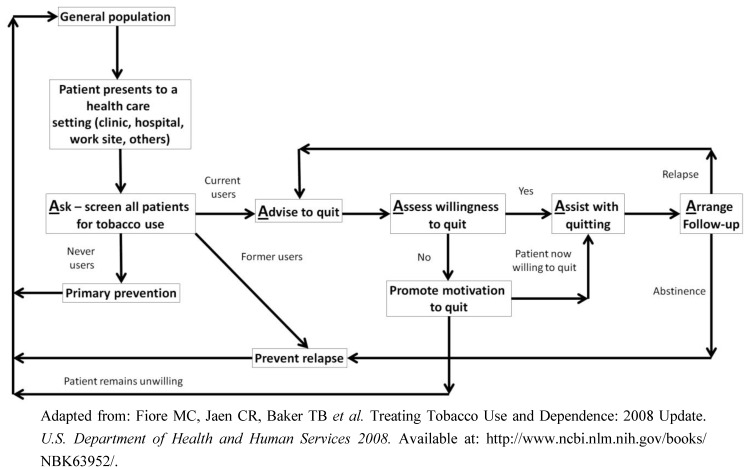
The 5 A’s of treating tobacco use and dependence.

The five A’s for treating tobacco use and dependence (Figure 2) can be used as a first step in addressing tobacco use with each clinical visit; **A**sk about tobacco use, **A**dvise to quit, **A**ssess willingness to quit, **A**ssist with quitting, and **A**rrange follow-up [126]. This is a strategy endorsed by the US Department of Health and Human Services to ensure that every patient who uses tobacco is identified, advised to quit and offered concrete tobacco cessation intervention. Among adolescents, a sixth A is usually included, to enable clinicians to **A**nticipate the risk of tobacco use among adolescent nonsmokers by assessing peer and parental influence and discussing possibility of tobacco initiation. Anticipation of this risk should be extended to all nonsmokers regardless of age.

## 9. Conclusions

HIV-infected persons have improved life expectancy in the HAART era, however, mortality rates remain higher than the general population and early-onset multimorbidity with non-AIDS defining illnesses is characteristic. Behavioral factors certainly contribute to this excess mortality and morbidity risk. The prevalence of tobacco use among HIV-infected persons is up to threefold higher than the general population. Due to shared pathogenic mechanisms with HIV infection, tobacco use magnifies the inherent risk of cardiovascular/peripheral vascular disease, ischemic stroke, lung and cervical cancer, and osteoporosis that HIV-infected persons have. 

HIV-infected adolescents, young adults and pregnant women are important targets for effective tobacco control programs. The cardiovascular and pulmonary risks associated with tobacco use are established early among adolescent smokers. Tobacco use during pregnancy also leads to a number of preventable maternal and fetal risks. At present, studies on effective tobacco control among these groups are lacking.

Addressing tobacco use early among HIV-infected adolescents, young adults, and pregnant women also provides a doable and practical preemptive strike against the tobacco epidemic in the HIV-infected adult population. Since these groups are usually engaged in HIV-related care, providers can repeatedly offer tobacco cessation counseling and follow-up on failed tobacco cessation attempts. Thus, it is of fundamental importance that HIV care providers actively assess tobacco use among their clinic population and that tobacco control strategies are routinely and consistently implemented in the outpatient clinic. 
